# Modeling the Effect of Milk Vetch–Rice Rotation on N Runoff Loss in the Middle and Lower Reaches of the Yangtze River

**DOI:** 10.3390/plants13223160

**Published:** 2024-11-10

**Authors:** Guodong Zhou, Cuilan Wei, Penghui Li, Hao Liang

**Affiliations:** 1 College of Agricultural Science and Engineering, Hohai University, Nanjing 210098, China; 221310040001@hhu.edu.cn (G.Z.); 231310040001@hhu.edu.cn (P.L.); 2 College of Environment and Ecology, Jiangsu Open University, Nanjing 210036, China; weicl@jsou.edu.cn; 3 College of Geography and Remote Sensing, Hohai University, Nanjing 210098, China; 4 Moith Agricultural Tech Co., Ltd., Chizhou 242800, China

**Keywords:** rice, green manure, nitrogen runoff loss, WHCNS-Rice model

## Abstract

The winter planting of green manure (GM) is widely used in South China to reduce chemical nitrogen (N) fertilizer use, improve soil fertility, and maintain rice yields, but its effect on N runoff loss in paddy fields remains unclear. This study combines multi-site field experiments with a process model (WHCNS-Rice) to assess how GM with reduced N fertilizer impacts N runoff loss and its forms in the Yangtze River’s middle and lower reaches, considering different rainfall years. The network field experiments included four treatments: conventional fertilization (FR), conventional fertilization plus straw return (FRS), GM with a 40% N reduction (MR), and GM-straw combined return with a 40% N reduction (MRS). Monitoring the results showed that compared to the winter fallow treatment, the GM treatments reduced the peak and average total N (TN) concentrations by 11.1–57.9% (average 26.9%) and 17.1–27.3% (average 22.3%), respectively. The TN runoff loss under the GM treatment decreased by 3.50–10.61 kg N ha^−1^ (22.5–42.1%). GM primarily reduced the runoff loss of dissolved inorganic N (DIN), with reductions at different sites ranging from 0.22 to 9.66 kg N ha^−1^ (8.4–43.4%), indicating GM effectively decreases N runoff by reducing DIN. Model simulations of ponding water depth, runoff, TN concentration in surface water, and TN loss in paddy fields produced the consistency indices and simulation efficiencies of 0.738–0.985, 0.737–0.986, 0.912–0.986, and 0.674–0.972, respectively, indicating that the model can be used to evaluate water consumption and N runoff loss in the GM-paddy system. The simulations showed that GM with a 40% N fertilizer significantly reduced N runoff loss under all rainfall conditions, with the greatest reductions in wet years. Under wet, normal, and dry conditions, the GM treatments significantly reduced average TN loss by 0.37–5.53 kg N ha^−1^ (12.77–29.17%), 0.21–5.32 kg N ha^−1^ (9.95–24.51%), and 0.02–3.2 kg N ha^−1^ (1.78–23.19%), respectively, compared to the winter fallow treatment. These results indicate that the combination of GM and a 40% reduction in N fertilizer can significantly reduce N runoff loss from paddy fields, demonstrating good effectiveness under various rainfall conditions, making it a green production model worth promoting.

## 1. Introduction

China is the largest producer and consumer of rice in the world, with annual production and consumption each accounting for 30% of the global total [1]. Nitrogen is the primary limiting factor for rice growth and development, and its application is a critical strategy to increase rice yields [2]. However, in the pursuit of high yields during rice production, farmers frequently apply excessive amounts of N fertilizer [3]. This not only increases input costs and reduces N fertilizer utilization efficiency but also results in significant nitrogen loss to water bodies via surface runoff, leaching, and drainage, causing nonpoint source pollution [4]. Multi-site monitoring in the Taihu Basin has revealed that the average total N runoff loss from paddy fields is 10.28 kg N ha^−1^ [5]. Another study estimated through models that the total N runoff loss from paddy fields in the middle and lower reaches of the Yangtze River is estimated at 0.24 Tg [6]. Nitrogen runoff from paddy fields is recognized as a major source of nonpoint source pollution [7].

Winter green manure is a traditional cropping practice in southern China’s paddy fields [8,9]. GM significantly contributes to improving soil fertility and productivity [8,10], enhancing soil health [11], and substituting for chemical fertilizers [12]. It is also considered as a promising approach to maintaining crop yields while reducing N fertilizer input and N loss [8]. Chinese milk vetch is a widely cultivated leguminous green manure in southern China [11,13]. The Chinese milk vetch–rice rotation system has been widely promoted and applied [10]. Long-term multi-site network observations in southern China’s paddy fields indicate that winter-sown Chinese milk vetch, when combined with 60% to 100% chemical fertilizer, not only ensures no yield reduction in rice but also enhances N fertilizer utilization efficiency by 6.6% to 31.1% [14,15,16,17]. Additionally, winter green manure can partially substitute for chemical fertilizers while regulating nutrient release rates, better aligning with the growth demands of rice, and demonstrating significant potential for reducing N loss [12]. Experiments in Zhejiang show that using Chinese milk vetch with reduced nitrogen fertilizer led to a decrease in N concentration in field water by 12.3% to 19.6% compared to conventional treatments [18]. Research in Anhui shows that compared to conventional fertilization, using Chinese milk vetch with a 30% reduction in N fertilizer resulted in reductions of 51% and 27.6% in ammonium N and total N runoff losses [19]. However, current research predominantly focuses on single-site studies and specific rainfall patterns without considering the significant impact of climatic conditions on regional paddy runoff loss. Therefore, further exploration is needed to investigate the impact of combining GM with a 40% reduction in N fertilizer on runoff N loss across a broader range and under different climatic conditions based on network monitoring.

Quantitative research on N loss in paddy fields is crucial for optimizing field management practices. However, long-term field experiments are time-consuming, labor-intensive, and difficult to generalize to broader regions. Integrating process-based models can effectively address this issue [20,21]. Commonly used paddy field ecosystem models include ORYZA3 [22], APSIM [23], DSSAT [24], DNDC [25], and WHCNS [26]. However, models specifically designed for GM practices are relatively rare. For example, Constantin et al. [27] used the LIXIM model to quantitatively assess the long-term impacts of continuous GM cultivation on N mineralization, crop N uptake, N use efficiency, and N leaching. The study found that GM can enhance soil N mineralization by 10 to 26 kg N ha^−1^ yr^−1^ and improve crop N absorption. Jasdeep et al. [28] used DNDC to predict the long-term effects of GM on crop yields, as well as soil organic carbon (SOC) and TN pools, finding that leguminous GM can increase cotton yield by 20 to 50 kg ha^−1^ yr^−1^ and enhance the SOC and TN content in the soil. Hung et al. [29] used the same model to predict that under future climate scenarios, planting GM could significantly reduce soil N_2_O emissions during the spring thaw period by 35 to 51 kg N ha^−1^ compared to winter fallow. Yin et al. [30] used the STICS model to calculate changes in soil N mineralization and N pathways over 34 years of crop rotation, showing that planting GM could increase soil N mineralization by 73 kg N ha^−1^ yr^−1^. Liang et al. [31] combined the WHCNS model with the LIXIM model to develop the WHCNS-Rice model, which is suitable for the GM-rice system. This model is better suited to China’s intensive management practices and has been validated and applied in multiple southern paddy fields [12]. This study hypothesizes that integrating GM with a 40% reduction in N fertilizer can significantly reduce N runoff loss under various climatic conditions. Unlike prior studies focused on a single site or specific rainfall events, with few utilizing models for related research, our research uses network monitoring and the WHCNS-Rice model to provide a comprehensive evaluation. The main objective is to validate the model’s capability to simulate N transfer processes and provide scientific support for promoting sustainable GM and N reduction strategies.

## 2. Materials and Methods

### 2.1. Site Description

The middle and lower reaches of the Yangtze River plain (27°33′–34°9′ N, 110°49′–122°30′ E) include the seven provinces (and municipalities) of Jiangsu, Anhui, Zhejiang, Jiangxi, Hubei, Hunan, and Shanghai [32]. The experiments were conducted at four test sites in the middle and lower reaches of the Yangtze River rice region. Nanjing station (31.65° N, 119.02° E) in Jiangsu Province belongs to the subtropical–warm transitional zone, and Chizhou station (30.65° N, 117.30° E) in Anhui Province, Gaoan station (28.42° N, 115.38° E) in Jiangxi Province, and Jingzhou station (30.33° N, 112.23° E) in Hubei Province are in the humid subtropical monsoon climate zone. The annual mean temperature (or precipitation) is 15.4 °C (1106 mm), 16.5 °C (1618 mm), 17.7 °C (1560 mm), and 16.6 °C (1168 mm) for Nanjing, Chizhou, Gaoan, and Jingzhou stations, respectively. The soils in all the stations are classified as stagnant anthrosols. The basic soil properties for these stations are listed in Table 1.

### 2.2. Experimental Design

The single rice–milk vetch cropping system is adopted in the Nanjing, Chizhou, and Jingzhou stations, while a rice–milk vetch double cropping system is implemented at the Gaoan station. The rice transplanting and harvesting times are shown in Table 1. Milk vetch was planted in late September or early October, then harvested and incorporated during its full flowering period. The field experiment in Nanjing was conducted in 2022, while those in Chizhou, Gaoan, and Jingzhou were performed in 2023.

The field experiment included four treatments: (1) conventional fertilization (FR); (2) conventional fertilization plus straw return (FRS); (3) GM with a 40% N reduction (MR); (4) GM-straw combined return with a 40% N reduction (MRS). Each treatment was replicated three times, resulting in a total of 12 plots, each with an area of 32 m^2^. The fertilization ratios were based on local farmers’ practices: at Gaoan and Nanjing stations, N was applied at a ratio of base fertilizer to tillering fertilizer of 6:4; at Chizhou station, the ratio was 11:7:2 for the base, tillering, and booting fertilizers; and at Jingzhou station, it was 7:2:1 for the same categories, with phosphorus and potassium fertilizers applied as a single base application. Fertilization amounts at the different experimental stations are shown in Table 1.

### 2.3. Observations and Measurement Methods

Surface water sampling: Water samples were collected on the day of fertilization (before fertilization) as Day 0, and then on Days 1, 3, 5, 7, and 10 after each fertilization. Sampling method: Using a medical syringe, the water samples were collected without disturbing the water layer following a diagonal sampling method. Five surface water samples were taken from each plot and mixed into a 200 mL sampling bag. The water samples were stored in refrigeration (at 4 °C) and analyzed within 48 h [18]. A high-frequency water level sensor was used to monitor the water level, and meteorological stations were installed in the field to collect meteorological and rainfall data.

Total nitrogen (TN) was measured using a TOC analyzer (TOC-L CPH, Shimadzu, Kyoto, Japan). The determination of dissolved total nitrogen (DTN) involved filtering the water samples through a 0.45 μm filter membrane and measuring with the same TOC analyzer. Ammonium nitrogen (NH_4_^+^-N) and nitrate nitrogen (NO_3_^−^-N) were measured using a continuous flow analyzer (SAN++, Skalar, Breda, the Netherlands) [33]. TN and PN were not measured at the Nanjing station. Particulate nitrogen (PN), dissolved inorganic nitrogen (DIN), and dissolved organic nitrogen (DON) were calculated using the difference method [34] as follows:

(1) DTN+PN=TN

(2) DIN+DON=DTN

(3) $${NH}_{4}^{+}-N+{NO}_{3}^{-}-N=DIN$$

### 2.4. WHCNS-Rice Model

The WHCNS-Rice model is a process-based crop model that can simulate water movement, N transport and transformation, and crop growth in paddy fields [26,35,36]. The model estimates crop evapotranspiration using the Penman–Monteith formula [37]. The infiltration and redistribution processes under unsaturated conditions are calculated using the Richards equation and the Green-Ampt model [38], while Darcy’s law is applied under saturated conditions [26,36]. The water depth at the field surface is calculated using a water balance approach, with the daily field water depth being

(4) $$Pd_{n}=Pd_{n-1}+P{rem}_{n}+{Irri}_{n}-{ET}_{n}-{Inf}_{n}-{Rf}_{n}$$

where *Pd_n_* and *Pd*_*n−*1_ are the ponding water depths at days *n* and *n*−1, respectively (mm); *Prem_n_*, *Irri_n_*, *ET_n_*, *Inf_n_*, and *Rf_n_* are the amounts of precipitation, irrigation, ET, infiltration, and runoff at day n, respectively (mm day^−1^); and *Pd_max_* is the maximum ponding water depth, (mm). Runoff (*Rf_n_*) can be computed by the following:

(5) $$P{Rf}_{n}=\left\{\begin{array}{cc} 0, & \;{Pd}_{n}<{Pd}_{max}\\ {Pd}_{n}-{Pd}_{max}, & \;{Pd}_{n}\geq {Pd}_{max} \end{array}\right.$$

The amount of runoff loss can be calculated by the following:

(6) $$Q=\sum_{i}^{n}\left(C_{i}\times {Rf}_{n}\times 10^{-2}\right)$$

where *Q* is the total runoff loss, (kg N ha^−1^), and *Ci* is the concentration of the runoff surface water quality for the *i*−th event, (mg L^−1^).

The model uses a convection–dispersion equation to describe the soil N migration process and references the DAISY model to describe the turnover of soil organic matter and the carbon-N cycle. The source–sink terms consider processes such as organic matter mineralization, biological immobilization, urea hydrolysis, ammonia volatilization, nitrification, denitrification, and crop uptake. For specific model principles, see the literature [31,36].

### 2.5. Calibration and Validation

Before model application, the relevant parameters (e.g., soil adsorption parameters, soil hydraulic parameters, nitrogen transformation parameters, and crop growth parameters) need to be calibrated and evaluated based on observed paddy field variables, including ponding water depth, runoff, nitrogen concentration, and yield. The ‘trial and error’ method was used to calibrate the model. This involved iteratively adjusting the parameters, running the model, comparing the simulation outputs with observations, and repeating the process until a close match was achieved. In this study, the model was calibrated using FR-treated paddy data for each site separately and validated with data from other treatments.

Three evaluation indices (*RMSE*, the root mean squared error; *d*, the index of agreement; and *E*, Nash–Sutcliffe efficiency) were used in model performance evaluation.

(7) $$MSE=\sqrt{\sum_{i=1}^{n}\frac{{\left(O_{i}-P_{i}\right)}^{2}}{n}}$$

(8) $$E=1-\frac{\sum_{i=1}^{n}{\left(O_{i}-P_{i}\right)}^{2}}{\sum_{i=1}^{n}{\left(O_{i}-\bar{O}\right)}^{2}}$$

(9) $$d=1-\frac{\sum_{i=1}^{n}{\left(O_{i}-P_{i}\right)}^{2}}{\sum_{i=1}^{n}{\left(\left|\left.P_{i}-O\right|\right.+\left|\left.O_{i}-O\right|\right.\right)}^{2}}$$

where *n* is the number of samples; *O_i_* and *P_i_* are the measured and simulated values at time *I*, respectively; and $\bar{O}$ is the mean of the measured data. *RMSE* is generally used to describe the mean absolute deviation between simulated and observed values, with smaller values indicating better simulation performance. The index *d* is another measure of the model’s simulation performance, ranging between 0 and 1, with values closer to 1 indicating better simulation results. The *E* value can be negative and is used to compare the deviation between simulated and observed values, with values closer to 1 indicating better simulation performance [35].

### 2.6. Scenario Analysis

**Figure 1:**
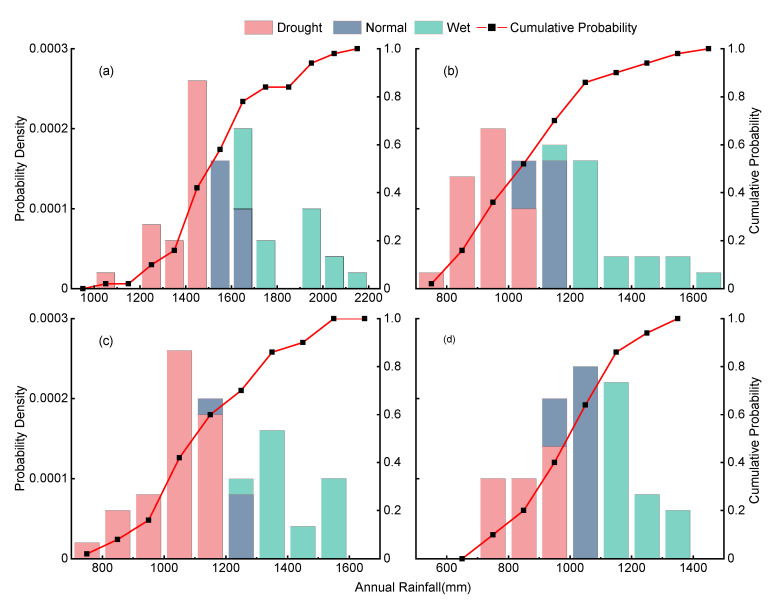
The 50-Year rainfall distribution and cumulative probability curves at Gaoan (**a**), Jingzhou (**b**), Nanjing (**c**), and Chizhou (**d**) stations.

The Chinese Weather Generator (NCC/GU-WG) uses a two-state first-order Markov chain method to generate dry and wet day sequences and employs a two-parameter GAMMA model to simulate precipitation amounts on wet days. Model parameters are calculated monthly to determine precipitation amounts for each month. The NCC/GU-WG simulates up to 671 stations, with model parameters estimated using 40 years of daily observed data from the corresponding stations, allowing for the random simulation of daily precipitation, maximum temperature, minimum temperature, and sunshine hours for a single station under various climate change scenarios [39]. This study uses NCC/GU-WG to predict daily precipitation for any 50-year period in the study area (Figure 1) and uses these results as input for the WHCNS-Rice model.

The 50 years of precipitation data are categorized based on the drought index (*DI*), where the calculation formula for *DI* is as follows:

(10) $$DI=\frac{P-A}{\sigma }$$

where *P*, *A*, and *σ* are the amounts of the fertility year rainfall, the mean multi-year rainfall, and the standard deviation of multi-year rainfall, respectively (mm). When *DI* > 0.35, it is considered a wet year; when −0.35 ≤ *DI* ≤ 0.35, it is considered an average year; and when *DI* < −0.35, it is considered a dry year [40,41].

## 3. Results

### 3.1. Dynamics of Different Forms of N in Field Water

**Figure 2:**
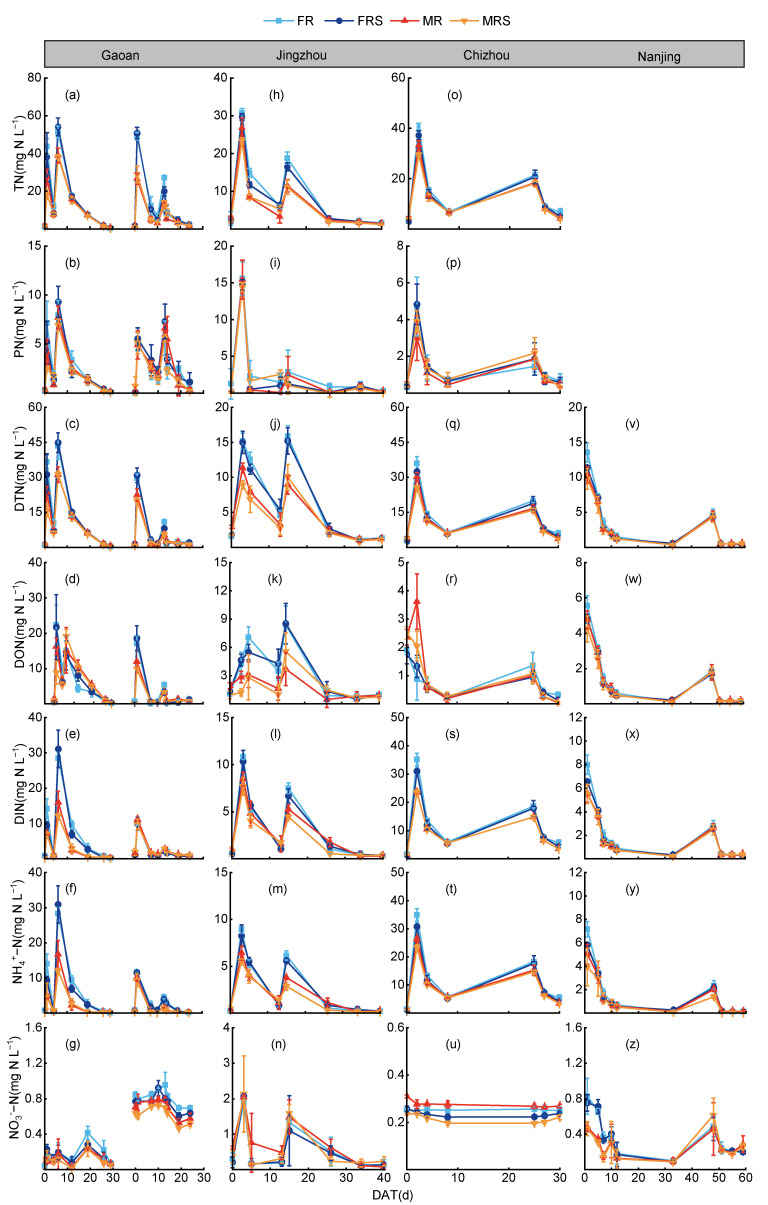
The dynamic changes in various N forms across the four regions: Gaoan (**a**−**g**), Jingzhou (**h**−**n**), Chizhou (**o**−**u**), and Nanjing (**v**−**z**). Panels a-g correspond to the dynamic changes in TN, PN, DTN, DON, DIN, NH_4_^+^−N, and NO_3_^−^−N. The same structure is followed for Jingzhou, Chizhou, and Nanjing. DAT: days after transplanting; FR: conventional fertilization; FRS: conventional fertilization plus straw return; MR: GM with a 40% N reduction; MRS: GM-straw combined return with a 40% N reduction.

After fertilization, the TN concentration in surface water increased sharply, peaking on the first day, then rapidly decreased and stabilized (Figure 2). Compared to the fallow treatment, the peak and average TN concentrations under the GM treatments were significantly reduced. Compared to the FR, FRS, and MR treatments, the peak TN concentration in the MRS treatment decreased by 24.1–57.9%, 20.7–50.7%, and 11.1–33.4%, respectively. After the application of tillering fertilizer, the TN concentration in all the treatments showed a similar trend of rising first, then falling, and finally stabilizing. The peak TN concentration in the MRS treatment was reduced by 15.3–41.1% and 12.3–32.9% compared to the FR and FRS treatments, respectively. The average TN concentration in the MRS treatment was reduced by 27.3% and 21.5% compared to the FR and FRS treatments, respectively (*p* < 0.01). The variation pattern of the PN concentration in the surface water was similar to that of the TN concentration, with the average PN concentrations ranked as FR > FRS > MR > MRS.

The concentration change patterns of DTN, DON, DIN, and NH_4_^+^-N in the surface water were similar to that of TN (Figure 2). Compared to the FR and FRS treatments, the average DTN concentration in the MR treatment decreased by 20.3% and 15.8%, respectively (*p* < 0.05), while that in the MRS treatment decreased by 26.1% and 21.4%, respectively (*p* < 0.05). Compared to conventional fertilization treatments, the GM treatments significantly reduced the average DTN concentration in the surface water, with the peak DTN concentration following the order FR > FRS > MR > MRS. The dynamic changes in the DTN concentration in the surface water were more gradual under the GM treatments compared to conventional fertilization treatments. The average DON concentration in the MRS treatment was significantly lower than in the FR and FRS treatments, with reductions of 22.2% and 19.8%, respectively (*p* < 0.01). The average DON concentration in the MR treatment decreased by 10.3% and 7.4% compared to the FR and FRS treatments, respectively. Compared to conventional treatments, the GM treatments reduced the DON content in the surface water and increased the DON/TN ratio. The average DIN concentration in the MRS treatment was significantly lower than in the FR and FRS treatments, with reductions of 31.8% and 24.5%, respectively (*p* < 0.01). The average DIN concentration in the MR treatment decreased by 26.5% and 18.9% compared to the FR and FRS treatments, respectively (*p* < 0.05) (Table 2). During the fertilization period, the NH_4_^+^-N/DIN ratio in all the treatments was generally over 90%. Compared to conventional treatments, the GM treatments significantly reduced the DIN content in the surface water, thereby reducing TN loss from the surface water.

### 3.2. Ponding Water Depth, Runoff and Nitrogen Concentrations

**Figure 3:**
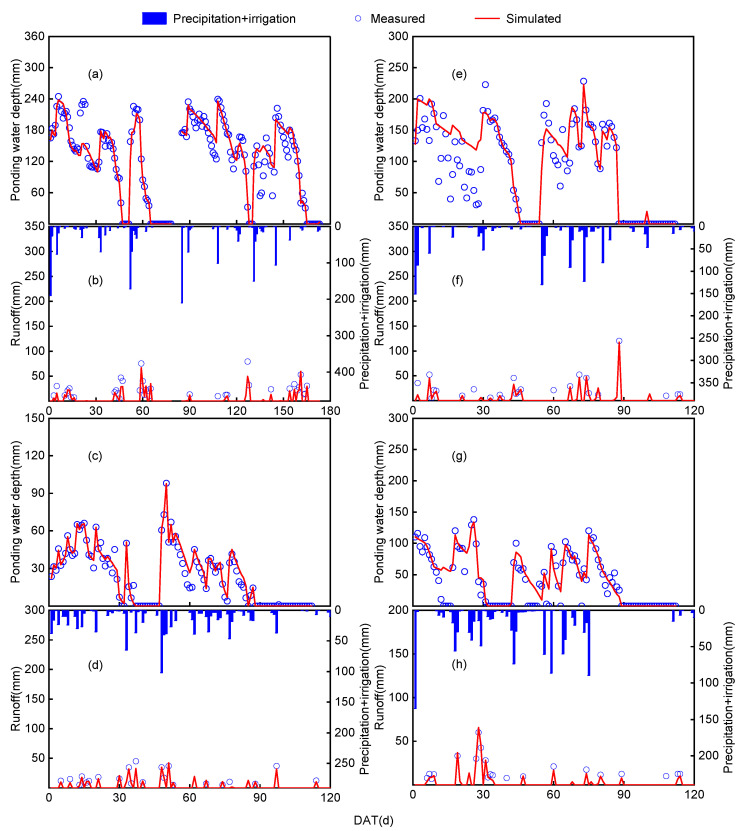
Comparison of measured and simulated values of ponding water depth and runoff at four sites: Gaoan (**a**,**b**), Nanjing (**c**,**d**), Jingzhou (**e**,**f**), and Chizhou (**g**,**h**). DAT: days after transplanting.

Except during the late tillering and preharvest drying periods, the surface water depth was controlled between 20 and 250 mm (Figure 3). According to model evaluation indices, the simulated surface water depth from the WHCNS-Rice model showed a high degree of agreement with the observed values. The *RMSE*, *d*, and *E* values for the simulated ponding water depth were 1.53–12.16 mm, 0.738–0.961, and 0.737–0.961, respectively, all within acceptable ranges (Table 3). Runoff usually occurred during significant rainfall or paddy drainage, and the simulated runoff peaks from the model matched the observed values well (Figure 3). According to the statistical indices, the *RMSE*, *d*, and *E* values for the simulated runoff were 0.119–4.68 mm, 0.949–0.985, and 0.951–0.986, respectively, all within acceptable ranges (Table 3). This indicates that the model can be used to quantify surface water levels and runoff losses in paddy fields across different regions.

**Figure 4:**
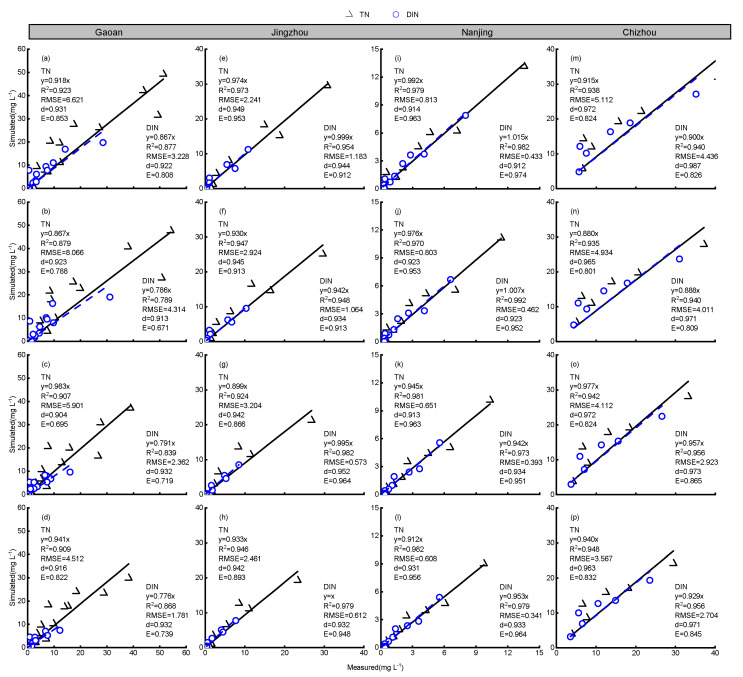
Simulation effects of the total nitrogen (TN)and dissolved inorganic nitrogen (DIN)at each experimental station. Figures (**a**–**d**) show the simulation results for Gaoan under the FR, FRS, MR, and MRS treatments. Similarly, figures (**e**–**h**) (Jingzhou), (**i**–**l**) (Nanjing), and (**m**–**p**) (Chizhou) present the simulation effects of each treatment on the total nitrogen (TN) and dissolved inorganic nitrogen (DIN) at each station. *RMSE*, root mean squared error; *d*, index of agreement; *E*, Nash–Sutcliffe efficiency.

The simulated N (TN and DIN) concentrations from the model showed a high degree of agreement with the observed values, with a coefficient of determination (R^2^) > 0.839, and the slope (β) of the linear regression equation was close to one (Figure 4). The RMSE, d, and E values for the simulated TN concentration in the surface water were 0.81–8.06 mg L^−1^, 0.90–0.97, and 0.69–0.96, respectively, while those for the simulated DIN concentration were 0.34–4.44 mg L^−1^, 0.91–0.98, and 0.67–0.97, all within acceptable ranges. This indicates that the model can be used to simulate N concentrations in surface water across different regions. Based on the model evaluation indices, the simulation performance for the DIN concentrations in the surface water was generally better than that for the TN concentrations.

### 3.3. Nitrogen Loss in Different Rainfall Years

**Figure 5:**
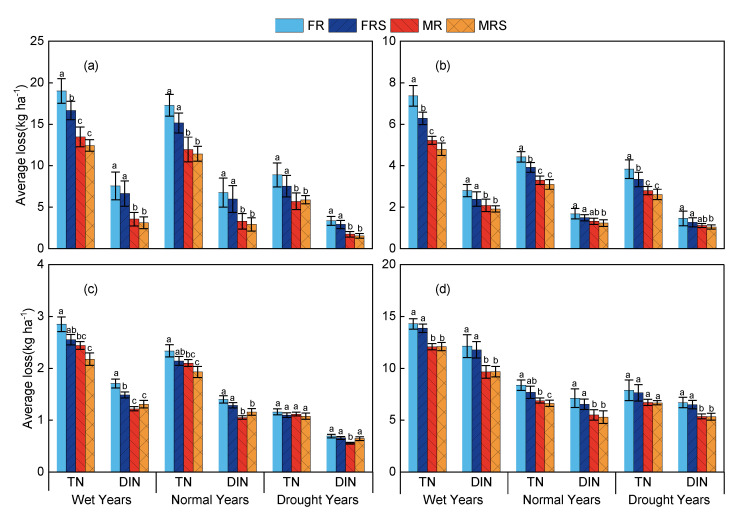
Mean nitrogen losses for different rainfall year types at Gaoan (**a**), Jingzhou (**b**), Nanjing (**c**), and Chizhou (**d**) stations. Different lowercase letters (**a**–**c**) above the bars indicate significant differences at the *P* < 0.05 level within the same treatment group.

In the three different rainfall year types, the average losses of TN and DIN followed the order FR > FRS > MR > MRS, with the highest N losses in wet years and the lowest in drought years (Figure 5). This indicates that the GM treatments effectively reduced the N runoff losses in paddy fields across all three rainfall year types. In wet years, the average TN loss in the GM treatments was significantly reduced by 12.77–29.17% (*p* < 0.05) compared to the fallow treatments, which was higher than in normal years (9.95–24.51%) and drought years (1.78–23.19%). In wet years, the average DIN loss in the GM treatments was significantly reduced by 12.07–53% (*p* < 0.05) compared to the fallow treatments, which was higher than in normal years (9.95–51.25%) and drought years (1.78–48.05%). The GM treatments reduced the N runoff losses across all the rainfall year types, with the best effects observed in wet years and relatively weaker effects in drought years.

The effect of combining GM with a 40% reduction in N fertilizer may vary significantly under different soil and climate conditions. In wet years, the GM treatments significantly reduced the N runoff losses at all the experimental stations. Gaoan station had the greatest reduction in N runoff losses. Compared to conventional treatments, the average TN and DIN runoff losses in the surface water under the GM treatments were significantly reduced by 25.3–29.17% and 52.95–53%, respectively. The emission reduction effects at Nanjing and Chizhou stations were relatively weaker, with the average TN and DIN runoff losses reduced by 12.77–15.50% and 12.07–28.60%, respectively (*p* < 0.05). In normal years, Gaoan station again showed the best emission reduction effect, with the average TN and DIN runoff losses significantly reduced by 3.71–5.32 kg N ha^−1^(24.51–30.79%) and 3.07–3.46 kg N ha^−1^ (51.14–51.25%), respectively. The emission reduction effect at Nanjing station was relatively moderate, with the average TN and DIN runoff losses reduced by 9.95–10% and 9.95–25%, respectively. In drought years, the average TN and DIN runoff losses at Gaoan station were reduced by 21.78–35.95% and 46.88–48.05%, respectively. In contrast, the average TN and DIN runoff losses at Nanjing station were reduced by 1.78–3.67% and 1.78–19.73%, respectively. Overall, the GM treatments at the different experimental stations showed significant emission reduction effects across different rainfall year types, with the effects being more pronounced under high rainfall conditions.

## 4. Discussion

### 4.1. Model Performance

The criteria for evaluating model performance often vary based on the simulated variables. Studies suggest that when the consistency index *d* ≥ 0.8, the simulation performance is considered very good; when *d* < 0.7, it is considered poor; and when d is between 0.7 and 0.8, it is considered good. The model efficiency *E* ranges from −∞ to 1, with values closer to 1 indicating better simulation performance [42,43]. According to these criteria, the simulation performance for variables such as the surface water depth, runoff volume, total N concentration, and DIN concentration in the surface water in this study was good, consistent with previous research results [44,45]. Gao et al. [46] reported that the N coefficient of determination (R^2^) was >0.68 when using ORYZA-N to simulate the coupling of water and N in paddy fields, whereas in this study, the N R^2^ was >0.84, indicating better simulation performance. This may be because the WHCNS-Rice model comprehensively considers the runoff process and soil N transformation. All the simulated indices in this study were within acceptable ranges, indicating the model’s suitability for simulating water and N transport processes in paddy fields under different management practices.

### 4.2. Effects of GM Substitution on N Losses

Nitrogen runoff losses in paddy fields are strongly correlated with fertilization management and runoff volume [47]. The first week following fertilization is particularly critical in controlling nitrogen losses [48,49]. In this study, N runoff losses were primarily observed in the early stages following fertilization. After fertilization, the N concentration in the surface water rose to a peak, and even with low runoff volume, significant N runoff losses still occurred. The primary form of N loss was DTN, dominated by DON and NH_4_^+^-N [50]. The GM treatments did not significantly affect the forms of N loss in paddy fields compared to conventional treatments [47]. This study found that the GM treatments significantly lowered peak N concentrations after fertilization, primarily by reducing N fertilizer input and lowering NH_4_^+^-N concentrations in surface water [51]. Additionally, the combination of Chinese milk vetch and reduced N fertilization optimized N supply and effectively promoted N uptake by rice [52,53]. Gao et al. [8] showed that the combination of Chinese milk vetch and a 40% reduction in N fertilization increased N uptake by 6.4% during the rice booting stage compared to conventional treatments. The combination of GM and chemical fertilizers significantly increased microbial populations [54]. GM also provided abundant organic matter [11], which promoted microbial reproduction and activity [8], accelerating the decomposition and transformation of DON and thus reducing DON concentrations in surface water. This study found that the DON concentration in the surface water was lower under the combined GM and straw return treatments compared to straw return alone. This could be because the combined GM and straw return decomposes quickly, releasing abundant available carbon and N, which promotes microbial utilization and expands the biological community [55]. Zhou et al. [9] showed that the combined return of organic materials optimizes nutrient structure and increases soil microbial biomass.

The combination of GM and reduced N fertilization not only increases and stabilizes yields but also effectively reduces N runoff losses in paddy fields [12]. Studies have shown that compared to conventional fertilization, the combination of Chinese milk vetch and a 20–30% reduction in N fertilization results in a reduction in TN runoff losses by 14–27.6% [19,56]. Previous studies have indicated that reductions in nitrogen fertilizer beyond 40% may lead to a decrease in rice yields [57]. Therefore, this study increased the N reduction ratio combined with Chinese milk vetch to 40%, and the results showed that the TN runoff losses were reduced by 28.4–45.6%. Under the conditions of maintaining yields, the higher the N reduction ratio, the greater the decrease in N runoff losses.

### 4.3. Effect of GM on N Runoff Loss Under Different Rainfall Years

The study observed that N losses and runoff volumes followed the following pattern: wet years > normal years > drought years, which is consistent with previous research results [58]. Given that the rice growing season often overlaps with the rainy season, surface runoff volumes tend to be higher in wet and normal years [59,60], making N more susceptible to loss in wet years [61]. This study found that the GM treatments significantly reduced the N runoff losses at all the experimental stations across the three different rainfall year types. The emission reduction effect was particularly notable in wet years, with reductions ranging from 12.77% to 29.17% (*p* < 0.05), higher than those observed in normal years (9.95–24.51%) and drought years (1.78–23.19%). In wet years, frequent and intense rainfall led to more frequent runoff events during high-risk N loss periods, thereby increasing the risk of N loss. This observation is consistent with the findings of Ren et al. [62]. N loss is closely related to runoff and N concentration in runoff [63]. The GM treatments effectively reduced the N concentrations in the surface water by decreasing N fertilizer input, significantly reducing the N runoff losses. In contrast, during drought years, runoff was primarily caused by active drainage from the fields, and the N concentrations were low across treatments with no significant differences, resulting in relatively weaker emission reduction effects from the GM treatments. The model simulations in this study confirmed that combining GM with a 40% reduction in N fertilization significantly reduced the N runoff losses in paddy fields, demonstrating the broad applicability of GM under various rainfall conditions. However, further studies are needed to assess the emission reduction effects of GM under extreme climate conditions.

## 5. Conclusions

This study integrated multi-site network observations and model simulations to quantify changes in both the forms and amounts of N runoff losses in paddy fields under GM treatments. The multi-site experiments showed that the peak and average TN concentrations under GM combined with reduced nitrogen fertilization were reduced by 11.1%-57.9% and 17.1–27.3%, respectively, compared to the fallow treatment. Additionally, the GM treatments significantly reduced the nitrogen runoff losses by 22.5–42.1%.

The combination of GM and reduced nitrogen fertilization primarily reduced the DIN content in runoff, thereby decreasing the N runoff losses (0.02–5.53 kg N ha^−1^) and lowering the risk of nonpoint source pollution. The model simulations confirmed that the GM treatments significantly reduced the N runoff losses in paddy fields across different rainfall year types, with particularly notable effects in reducing DIN losses (0.01–4.01 kg N ha^−1^). This indicates that GM not only showed excellent emission reduction effects in field trials but also was further validated by the model simulations to have broad applicability and effectiveness under different climate conditions.

## Figures and Tables

**Table 1:** Basic soil properties and N inputs at different experimental stations.

Experimental Stations	Longitude (°)	Latitude (°)	Transplanting Date	Harvest Date	Conventional N Application (kg ha^−1^)	Basic Soil Properties					Texture (USDA)
						PH	SOC (g kg^−1^)	TN (g kg^−1^)	AP (mg kg^−1^)	AK (mg kg^−1^)	
Gaoan	115.38	28.42	4.26(7.24)	7.17(10.20)	150(180)	5.82	23.49	0.99	12.51	49.78	Silty clay
Jingzhou	112.23	30.33	5.20	9.06	165	7.06	22.00	2.00	10.60	167.00	Silty clay
Nanjing	119.02	31.65	6.11	10.01	200	6.23	24.44	1.47	20.44	107.25	Silty clay loam
Chizhou	117.30	30.65	6.06	9.26	192	6.27	27.64	1.30	13.14	62.39	Clay

Note: SOC: soil organic matter; TN: total nitrogen; AP: available phosphorus; AK: available potassium. The contents of the table in parentheses are the corresponding data for late rice.

**Table 2:** Reduction rates of nitrogen indicators in MR and MRS treatments.

Indicator	Reduction in MR vs. FR (%)	Reduction in MR vs. FRS (%)	Reduction in MRS vs. FR (%)	Reduction in MRS vs. FRS (%)
DTN	20.3	15.8	26.1	21.4
DON	10.3	7.4	22.2	19.8
DIN	26.5	18.9	31.8	24.5

Note: FR: conventional fertilization; FRS: conventional fertilization plus straw return; MR: GM with a 40% N reduction; MRS: GM-straw combined return with a 40% N reduction.

**Table 3:** Model performance in simulating ponding water depth, runoff, and yield.

	Index	Gaoan	Jingzhou	Nanjing	Chizhou
Ponding water depth (mm)	*RMSE*	5.260	12.160	1.530	7.010
	*d*	0.933	0.796	0.961	0.738
	*E*	0.932	0.795	0.961	0.737
Runoff (mm)	*RMSE*	4.680	2.180	1.550	0.119
	*d*	0.949	0.977	0.961	0.985
	*E*	0.951	0.977	0.963	0.986
Yield (kg ha^−1^)	*RMSE*	191.03	202.01	236.46	160.93
	*d*	0.993	0.972	0.995	0.986
	*E*	0.992	0.956	0.984	0.967

Note: *RMSE*, root mean squared error; *d*, index of agreement; *E*, Nash–Sutcliffe efficiency.

## Data Availability

The datasets generated for this study are available upon request to the corresponding author.
